# Abnormal MEG Oscillatory Activity during Visual Processing in the Prefrontal Cortices and Frontal Eye-Fields of the Aging HIV Brain

**DOI:** 10.1371/journal.pone.0066241

**Published:** 2013-06-20

**Authors:** Tony W. Wilson, Howard S. Fox, Kevin R. Robertson, Uriel Sandkovsky, Jennifer O’Neill, Elizabeth Heinrichs-Graham, Nichole L. Knott, Susan Swindells

**Affiliations:** 1 Department of Pharmacology and Experimental Neuroscience, University of Nebraska Medical Center, Omaha, Nebraska, United States of America; 2 Department of Neurological Sciences, University of Nebraska Medical Center, Omaha, Nebraska, United States of America; 3 Department of Internal Medicine, University of Nebraska Medical Center, Omaha, Nebraska, United States of America; 4 Center for Magnetoencephalography, University of Nebraska Medical Center, Omaha, Nebraska, United States of America; 5 Department of Neurology, University of North Carolina School of Medicine, Chapel Hill, North Carolina, United States of America; 6 Department of Psychology, University of Nebraska-Omaha, Omaha, Nebraska, United States of America; Wake Forest School of Medicine, United States of America

## Abstract

**Objective:**

Shortly after infection, HIV enters the brain and causes widespread inflammation and neuronal damage, which ultimately leads to neuropsychological impairments. Despite a large body of neuroscience and imaging studies, the pathophysiology of these HIV-associated neurocognitive disorders (HAND) remains unresolved. Previous neuroimaging studies have shown greater activation in HIV-infected patients during strenuous tasks in frontal and parietal cortices, and less activation in the primary sensory cortices during rest and sensory stimulation.

**Methods:**

High-density magnetoencephalography (MEG) was utilized to evaluate the basic neurophysiology underlying attentive, visual processing in older HIV-infected adults and a matched non-infected control group. Unlike other neuroimaging methods, MEG is a direct measure of neural activity that is not tied to brain metabolism or hemodynamic responses. During MEG, participants fixated on a centrally-presented crosshair while intermittent visual stimulation appeared in their top-right visual-field quadrant. All MEG data was imaged in the time-frequency domain using beamforming.

**Results:**

Uninfected controls had increased neuronal synchronization in the 6–12 Hz range within the right dorsolateral prefrontal cortex, right frontal eye-fields, and the posterior cingulate. Conversely, HIV-infected patients exhibited decreased synchrony in these same neural regions, and the magnitude of these decreases was correlated with neuropsychological performance in several cortical association regions.

**Conclusions:**

MEG-based imaging holds potential as a noninvasive biomarker for HIV-related neuronal dysfunction, and may help identify patients who have or may develop HAND. Reduced synchronization of neural populations in the association cortices was strongly linked to cognitive dysfunction, and likely reflects the impact of HIV on neuronal and neuropsychological health.

## Introduction

Cognitive impairments are a recognized complication observed in 35–70% of persons infected with human immunodeficiency virus type one (HIV-1), including those who are both treated and untreated [1]–[5]. While combination antiretroviral therapy (cART) has robustly decreased the prevalence of HIV-associated dementia (HAD), milder forms of HIV-associated neurocognitive disorders (HAND) remain prevalent [1]–[3], [5], [6]. Despite such high prevalence, a differential diagnosis can often be difficult, requiring neurocognitive testing and exclusion of opportunistic infections, psychiatric disorders, drug toxicities, brain tumors, and other factors to rule out other causes. Notably, there are currently no diagnostic tests or any specific biomarkers that can precisely pinpoint HAND. Likewise, there are no specific tests or biomarkers that can be utilized to measure disease progression or to assess therapeutic responses.

To further understanding of the pathophysiology, numerous neuroimaging studies have evaluated HIV-infected patients with and without HAND. These studies have included structural and functional magnetic resonance imaging (fMRI), magnetic resonance spectroscopy (MRS), and other imaging techniques. Some of the earliest neuroimaging studies of HAND utilized MRS and found widespread evidence of aberrant metabolite levels suggesting severe inflammation and neural loss at all stages of infection [7]–[11]. Several fMRI studies have investigated neural activation during attention and working memory tasks in HIV-infected patients with and without cognitive impairment [12]–[16]. These studies have shown that HIV-infected patients exhibit greater activation and/or larger load-dependent increases in activation within the frontal and parietal cortices serving task performance [12]–[16]. Interestingly, outside of the association cortices, the opposite pattern (HIV<controls) may exist in the primary sensory cortices. Two recent studies showed decreased activation in the primary visual cortices during a basic visual stimulation task using fMRI, and reduced resting cerebral-blood-flow to the same visual regions in HIV-infected patients with and without cognitive deficits relative to uninfected controls [17], [18]. These two studies did not evaluate cortical areas outside of the occipital region, which complicates any direct comparison to earlier studies of working memory and attention.

While fMRI studies have been especially informative in this area, there is at least preliminary evidence that neurovascular coupling is abnormal in patients with HIV infection [19]. One recent study reported significantly lower test-retest reliability of fMRI’s blood oxygenation-level-dependant (or BOLD) signal in HIV-infected compared with uninfected persons [19]. Such findings are concerning as the faithfulness of the fMRI signal as an indirect index of neuronal activity depends on the integrity of neurovascular coupling. In this study, we utilize magnetoencephalography (MEG) to evaluate cortical activity during visual processing in older HIV-infected patients and a matched group of uninfected controls. MEG is a direct measure of neurophysiological activity, which quantifies the ultra-minute magnetic fields that naturally emanate from postsynaptic electrical currents within populations of active pyramidal cells in the cortex. The method is completely noninvasive, possesses millisecond temporal resolution and good spatial precision, and is entirely unaffected by abnormalities in neurovascular coupling. We evaluated an older group of patients (i.e., ∼60 years-old) because HIV-infected persons are living much longer in the cART era, and these patients are an underrepresented group in research studies. Our primary focus was activation differences between infected and uninfected persons in both the primary visual cortices and higher-level association cortices during visual processing. We hypothesized that HIV-infected patients would exhibit reduced activation in modality-specific occipital regions and greater or equal activation in the association cortices.

## Methods

We evaluated 12 HIV-infected adults (3 females) and 11 uninfected healthy controls (3 females). Mean age was 57.92 years (range: 50–69 years) in the HIV-infected group, and 59.36 years (range: 53–70 years) in the control group. Controls were individually-matched to patients in regards to age, sex, ethnicity, and handedness. HIV-infected participants were receiving effective antiretroviral therapy and had undetectable viremia. The mean duration of HIV diagnosis was 17 years (range: 10–22), and the average CD4+ T-cell count was 741 cells/mm^3^ (range: 267–1276). Exclusionary criteria included any pre-existing major psychiatric or neurological disorder, active brain infection (except HIV-1), presence of brain neoplasm or space-occupying lesion, history of head trauma, current substance abuse, and the MEG Laboratory’s standard exclusion criteria (e.g., dental braces, metal implants, battery operated implants, and/or any type of ferromagnetic implanted material). Written informed consent was obtained following the guidelines of the University of Nebraska Medical Center’s Institutional Review Board, which approved the study protocol.

### Neuropsychological Assessments

All patients underwent a battery of neuropsychological testing. This neuropsychological battery was sensitive, tested multiple domains, yet was relatively brief and convenient, and adhered to the recommendations of the Frascati consensus [5]. The battery assessed multiple functional domains including, gross motor (timed gait), fine motor (grooved pegboard), language (WRAT 4 reading), verbal learning (Hopkins Verbal Learning Test – Revised), verbal memory (HVLT-R, delayed recall), speed of processing (Trailmaking-A, digit symbol), attention and working memory (Paced Auditory Serial Addition Task), and executive functioning (verbal fluency, Stroop, and Trailmaking-B). Correlation analyses were conducted on a subset of these neuropsychological metrics and the MEG data (see below). Selection of this subset was based on the distribution of scores across patients and the cognitive faculty probed by individual measures.

### Experimental Paradigm

Throughout the visual stimulation paradigm, participants were seated within the magnetically-shielded room with both arms resting on a tray attached to the chair body. Participants were instructed to remain still and fixate on a centrally-presented cross hair, while a small checkerboard-pattern square was presented in the top-right visual quadrant. The stimuli were of 67 ms duration and were presented with a variable inter-stimulus interval of 2.1–3.2 s. A total of 120–130 trials were collected per participant.

### MEG Data Acquisition & Coregistration with Structural MRI

With an acquisition bandwidth of 0.1–330 Hz, neuromagnetic responses were sampled continuously at 1 kHz using an Elekta Neuromag system (Helsinki, Finland) with 306 MEG sensors. During data acquisition, participants were monitored via real-time audio-video feeds from inside the magnetically-shielded room. Each MEG data set was individually corrected for head motion during the recording (offline), and subjected to noise reduction using the signal-space separation method with a temporal extension [20]. Each participant’s MEG data was then coregistered with structural Teighted MRI data. All structural MRI data were aligned parallel to the anterior and posterior commissures and transformed into the Talairach coordinate system [21] using BrainVoyager QX (Brain Innovations, The Netherlands).

### MEG Pre-Processing

Artifact rejection was based on a fixed threshold method, supplemented with visual inspection. Epochs were of 1.4 s duration (−0.4 to 1.0 s), with 0.0 s defined as the stimulus onset and the baseline being the −0.4 to 0 s window. Artifact-free epochs were transformed into the time-frequency domain using complex demodulation [22], and the resulting spectral power estimations per sensor were averaged over trials to generate time-frequency plots of mean spectral density. These data were then normalized to allow task-related power fluctuations to be visualized in sensor space as event-related synchronizations (ERS; power increases) and desynchronizations (ERD; power decreases).

### MEG Time-Frequency Statistics

The specific time-frequency windows used for imaging were determined by statistical analysis of the spectrograms corresponding to the MEG gradiometer sensors. Essentially, each data point in the spectrogram was evaluated for group differences using a mass univariate approach based on the general linear model. We conducted the analysis in this way, and not using one-sample t-tests (i.e., across both groups), because we were interested in potential oscillatory differences between groups on parameters such as response duration or peak frequency. Such effects could easily wash out in a one-sample t-test, as this would essentially show the overlapping bins in time-frequency space (across groups). To reduce the risk of false positive results while maintaining reasonable sensitivity, a two stage procedure was followed to control for Type 1 error. In stage one, two-sample t-tests were conducted on each data point and the output spectrogram of t-values was thresholded at (p<0.05) to define time-frequency bins containing potentially significant differences in oscillatory activity. In stage two, time-frequency bins that survived the (p<0.05) threshold were clustered with temporally or spectrally neighboring bins that were also above the (p<0.05) threshold, and a cluster value was derived by summing the t-values of all data points in the cluster. Nonparametric permutation testing was then used to derive a distribution of cluster-values, and the significance level of the observed clusters (from stage one) was tested using this distribution [23], [24]. For each comparison, 10,000 permutations were computed to build a distribution of cluster values.

### MEG Source Imaging

Cortical networks were imaged through an extension of the linearly-constrained minimum variance vector beamformer [25], which employs spatial filters in the frequency domain to calculate source power for the entire brain volume. Following convention, the source power in these images was normalized per subject using a separately averaged pre-stimulus noise period of equal duration and bandwidth [26]. In principle, the beamformer operator generates a spatial filter for each grid point, which passes signals without attenuation from the given neural region while minimizing interference from activity in all other brain areas. The properties of these filters are determined from the MEG covariance matrix and the forward solution for each grid point in the image space, which are used to allocate sensitivity weights to each sensor in the array for each voxel in the brain (for a review, see [27]).

Normalized source power was computed for the time-frequency range of interest per participant at 4.0×4.0×4.0 mm resolution. Each subject’s functional images, which were co-registered to anatomical images prior to beamforming, were transformed into a standardized space [21] using the transform previously applied to the structural MRI volume. MEG pre-processing and imaging used the Brain Electrical Source Analysis (BESA version 5.3.2) software, and MEG-MRI coregistration used BrainVoyager QX (Version 2.2).

The effect of group was examined using a random effects analysis for the time-frequency bin of interest, while one-sample *t*-tests were conducted to probe activation patterns in each group. To reduce the risk of false-positive results, we followed a procedure analogous to the time-frequency analyses described above. Briefly, statistical parametric maps were thresholded at (p<0.01) to define clusters of potentially significant activation. Permutation testing was used to derive a distribution of cluster-values and the significance level of the observed clusters was tested directly using this distribution [23], [24]. For each comparison, 10,000 permutations were computed to build a distribution of cluster values.

## Results

**Figure 1:**
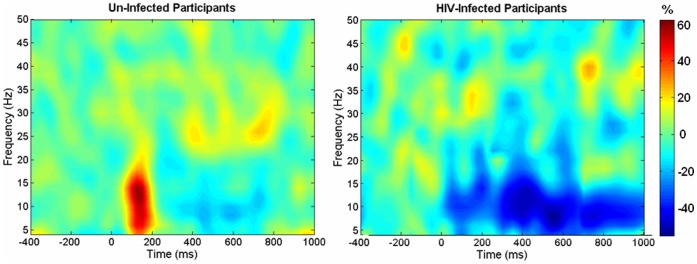
Average Time-Frequency Spectra in Un-Infected Controls and HIV-Infected Patients. Time (in ms) is denoted on the x-axis, with 0 ms defined as the onset of visual stimulation and the baseline being the −400 to 0 ms time period. Frequency (in Hz) is shown on the y-axis. The average spectra for a MEG gradiometer in the right frontal area, expressed as percent difference from baseline (scale bar appears on far right side), are shown for un-infected participants on the left and for HIV-infected patients on the right. The early 6–12 Hz synchronization can be easily discerned in controls, whereas patients exhibited a slight desynchronization during this time period. In contrast, after 300 ms both groups exhibited desynchronization in the 6–12 Hz range, with HIV-infected patients showing a slightly stronger desynchronization on average, although this response was highly variable across participants and did not approach significance.

Statistical analysis of sensor-level time-frequency spectrograms indicated significant event-related oscillatory differences in the 6–12 Hz band during the 50–225 ms post-stimulus time window (p<0.05, corrected; see Figure 1). These responses were concentrated in frontal aspects of the array. We selected a time-frequency window of equal bandwidth and duration from the baseline period (6–12 Hz, −175 to 0 ms), and imaged these oscillatory changes using beamforming.

### Task Effects

Uninfected controls exhibited significant ERS responses in the 6–12 Hz range between 50–225 ms in the right dorsolateral prefrontal cortex (DLPFC), the inferior surface of the left calcarine fissure extending onto the left inferior occipital cortices, right (and slightly left) frontal eye-fields (FEF) near the supplementary motor area, and the posterior cingulate cortices (all p’s <0.01, corrected). In contrast, HIV-infected participants showed significant ERD responses in the 6–12 Hz band within the right DLPFC and the right FEF (p<0.01, corrected). In addition, HIV patients exhibited ERS in the left inferior occipital cortices (p<0.01, corrected).

### Group Effects

**Figure 2:**
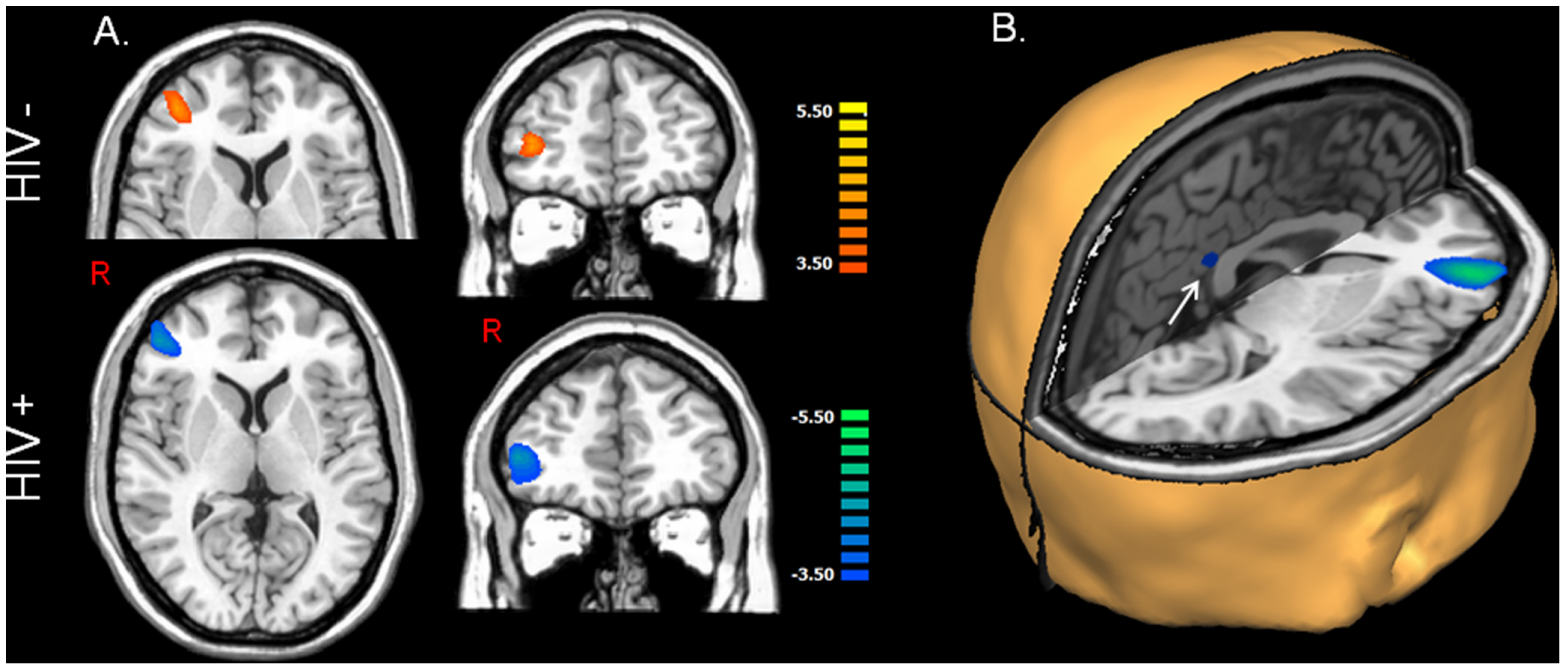
Dorsolateral Prefrontal Cortex (DLPFC) Activity during Visual Processing. (A) Group statistical maps (p<0.01) show strong ERS responses in the right DLPFC of uninfected controls (top row) and strong ERD activity in the same neural regions of HIV-infected patients (bottom row). Thus, visual stimulation elicited activity in opposite directions within the DLPFC and other regions of patients and controls. Color scale bars indicate t-statistical values. (B) A -rendition showing neural regions where significant (p<0.01, corrected) group differences were detected during visual processing. As shown, HIV-infected patients exhibited strong reductions in neuronal responses between 6–12 Hz in the right DLPFC, right frontal eye fields (not shown), and a small area of the inferior posterior cingulate. All images are shown in radiological convention (R = L). Blue: ERD, Orange: ERS.

**Figure 3:**
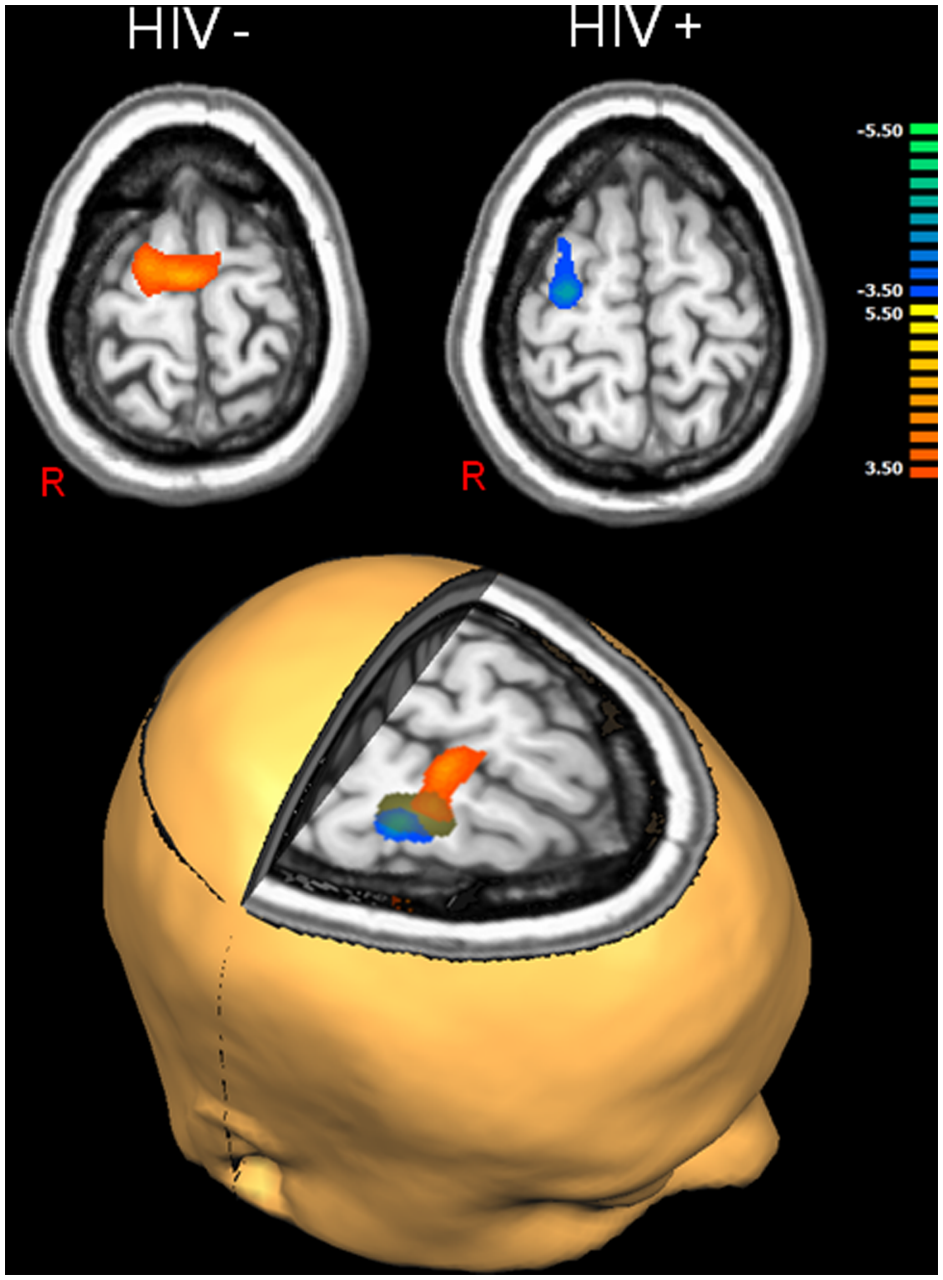
MEG Responses in the Frontal Eye-Fields (FEF). (A) axial images showing the maximum response in uninfected controls (top left) and HIV-infected patients (top right). Consistent with the right dorsolateral prefrontal cortex findings, HIV-infected patients exhibited significant ERD in the right FEF in response to the visual stimulation, whereas uninfected controls exhibited strong ERS that was slightly more midline focused. The rendition (bottom) shows the ERS (orange) found in controls, the ERD (blue) found in patients, and the FEF cortical region where significant group differences were found (green) superimposed on the same standardized brain. All maps have been thresholded at (p<0.01, corrected) and the images are displayed in radiological convention (R = L). The color scale bar shows t-statistical values.

Group-mean ERS responses in the modality-specific occipital cortices were stronger in the control group, but this effect did not approach significance. In contrast, the groups did differ in the right DLPFC and FEF (p<0.01, corrected), as HIV-infected patients exhibited strong decreases (i.e., ERD) and controls showed robust increases (ERS) in these neural areas during the 50–225 ms window (see Figures 2–3). Finally, controls had significantly stronger ERS responses in the posterior cingulate region.

### Neuropsychological & MEG Correlations

To evaluate possible relationships between neuropsychological functioning and regional MEG activity in HIV-infected patients, we conducted a series of exploratory Pearson-correlations using the maximum amplitude value in each brain region where group differences were found, and the patient scores on individual neuropsychological assessments. These analyses indicated that the retention index of the Hopkins Verbal Learning Test Revised (HVLT-R) was positively correlated with the amplitude of neural activity in the right FEF, *r*(12) = 0.72 (*p*<0.05, Bonferroni-corrected), and the right DLPFC, *r*(12) = 0.76 (*p*<0.05, Bonferroni-corrected). These correlations showed that patients with weaker ERD responses (closer to controls) had higher retention scores. The other neuropsychological tests that we examined were not significantly correlated with MEG activity.

## Discussion

We evaluated neurophysiological activity during basic visual stimulation in HIV-infected older adults and a matched sample of uninfected healthy controls using MEG imaging. Our most important finding was that visual stimulation induced oscillatory neuronal responses of opposite direction in the association cortices of patients and controls. While between-group activation differences were expected in these brain regions, such a pronounced effect was surprising. Furthermore, neural activity in the right FEF and DLPFC (i.e., association areas) were correlated with neuropsychological performance in HIV-infected patients, thereby linking these aberrations in brain activity to impairments in cognitive functioning. Beyond the association cortices, we hypothesized that HIV-infected patients would exhibit reduced activation in modality-specific occipital regions relative to controls, but this effect did not approach significance. Below, we discuss the implications of these findings for the neural basis of HAND.

In this study, HIV-infected patients showed decreased neural synchronization (i.e., ERD) during visual processing in neural populations within the right DLPFC and FEF. Conversely, uninfected controls showed increased neural synchronization (i.e., ERS) in these same brain regions. Interestingly, the amplitude of the ERD in these two brain areas was correlated with scores on the retention index of the HVLT-R in HIV-infected patients, which is a test known to be sensitive to HAND [28], [29]. Essentially, those patients with the strongest ERD had the lowest retention rates and those with the smallest ERD (or a slight ERS) had the highest retention rates, which makes sense given the pattern of strong ERS responses in uninfected controls. Previous parametric fMRI studies of working memory and attention have shown that uninfected controls exhibit stronger activation than patients in the right prefrontal cortices and the FEF during the easiest condition (e.g., lowest memory or attention load), but that this pattern reverses (patients>controls) as the memory load increases [13], [14]. Such observations are in line with the current findings and may suggest a failure to orient to the task, or more simply that the brain is less sensitive or less responsive to low-level stimulation that does not require extensive attentional resources. The basic visual stimulation task used in the current study only required that participants focus on a centrally-presented crosshair, and avoid saccades to the visual stimulus that intermittingly appeared in the participant’s top-right visual quadrant. Thus, ERS activity detected in the right DLPFC and FEF of controls potentially reflects neural activity serving low-level attentional control (i.e., right DLPFC) of processes that maintain or control eye-position in the visual field. Finally, Thompson et al. showed that the degree of cognitive impairment in non-demented HIV-infected patients was strongly correlated with the thickness of the DLPFC [30].

HIV-infected patients also exhibited decreased ERS in inferior regions of the posterior cingulate relative to controls. The posterior cingulate is known to be a critical node in the default-mode network, which is a group of brain areas that are more active during awake rest than during cognitively active states and that maintain strong inter-regional coupling [31]–[33]. A large corpus of normative fMRI studies have identified and characterized this network, which includes medial prefrontal cortex, the posterior cingulate/precuneus cortices, and the mediolateral inferior parietal cortices bilaterally [34]–[36]. Interestingly, an EEG/fMRI study of the default-mode network showed that 2–9 Hz activity was inversely correlated with BOLD measurements [37], and a recent intracranial EEG study linked a similar neuronal response rate (i.e., frequency band) with activity in the default-mode network [38]. Moreover, recent methodological advancements have allowed characterization of the brain's resting-state networks with spatially-resolved MEG [39]. Brookes et al. [39] suggested that the vast majority of resting-state networks detected using fMRI correspond to beta-frequency activity in the MEG signal. However, one critical exception was the default-mode network, which was strongest in the alpha-frequency range (8–13 Hz) in their study of ten healthy adults [39]. Thus, the greater ERS in the posterior cingulate region likely reflects enhanced suppression of default-mode activity in controls during the visual processing task.

Across the DLPFC, FEF, and posterior cingulate HIV-infected patients exhibited reduced neural synchronization in the 6–12 Hz frequency range. This frequency band includes part of the classic theta (4–7 Hz) and alpha bands (8–12 Hz), which have been linked to memory function (theta and alpha), visual processing (alpha), and many other cognitive functions. In particular, frontal theta activity has been linked to working memory and visual attention processes [40]–[42]. In addition, several studies have shown that frontal theta increases are correlated with memory load, in that larger memory loads (e.g., 5 items compared 3 items) are associated with stronger frontal theta activity [40]–[41], [43]–[44]. There is also some evidence that frontal alpha activity increases with memory load [42], although these reports have been less frequent. More commonly, alpha activity has been linked to active inhibition of task irrelevant brain regions during attention and working memory tasks [45]–[46], with some data further suggesting that alpha-frequency activity may be a critical mechanism for overall network coordination during cognitive processing [42], [46]. In the current study, we observed classic alpha desynchronization in occipital cortices (i.e., 6–12 Hz) following visual stimulation in both groups, which would be consistent with a release of occipital inhibition to allow active visual processing. We also observed increased 6–12 Hz activity in the right DLPFC and FEF in controls relative to HIV-infected patients. Such increased alpha activity is consistent with other studies that used visual attention tasks, and may suggest that the increased frontal alpha activity in controls reflects enhanced attentional processing relative to patients [42].

In contrast to several recent reports, we did not observe hypo-activation in the primary visual regions of HIV-infected patients [17], [18]. Previous studies have shown decreased regional cerebral blood flow (CBF) in the primary visual cortices during rest, and reduced fMRI BOLD activation in occipital cortices during visual stimulation. The current study did observe greater activation (marginal means) in the left occipital region in uninfected controls, but this effect did not approach significance. This difference between studies may reflect the substantially larger sample size used by Ances et al., as these studies had more than twice as many participants per group than the current study and thereby, greater sensitivity to detect smaller effects [17], [18]. However, Ances and colleagues also recently showed that the test-retest reliability of fMRI (BOLD and CBF) measures in HIV-infected patients are much lower than controls, and that the intrinsic coupling between oxygenation and blood flow is aberrant in HIV-infected patients [19]. Such a breakdown in the coupling of these parameters could produce spurious results and lead to inconsistent findings between fMRI and MEG studies. Unfortunately, these studies only investigated the occipital area alone [17], [19] or the lenticular nuclei and the occipital region [18]. Thus, it is impossible to discern whether these previous fMRI studies would have also detected the larger group effects that we observed in the right DLPFC, right FEF, and posterior cingulate. Essentially, we found clear statistical differences in these regions with a much smaller sample, and the corresponding effect sizes were of course much larger than those seen in visual cortices.

Previous human imaging studies using fMRI, MRS, and other techniques have improved understanding of the neural bases of HAND, but thus far MEG imaging has had an extremely limited role in this area. To our knowledge, only two previous MEG studies have investigated patients with HIV/AIDS. One reported aberrant mutual information between sensors in the anterior right part of the MEG helmet and those in the posterior left part. Measures of mutual information are closely related to functional connectivity, thus these findings suggest abnormal functional connectivity between right frontal and left posterior regions in HIV-infected patients [47]. Although some caution is warranted as MEG sensor-level data (i.e., magnetic field strength measurements) contains neural activity from a mix of many distinct brain areas, and it is tenuous to link such activity to particular brain regions. The other MEG study examined the reliability of MEG measurement, and provided preliminary evidence that broadband sensor-level data had good test-retest reliability after ∼24 weeks in both HIV-infected patients and controls [48]. Such findings are clearly supportive of using MEG to develop biomarkers for the early identification of HAND, and the current study also makes important contributions to this long-term goal.

To close, we evaluated whole-brain neurophysiological activity using MEG in HIV-infected patients and a matched group of uninfected controls who were performing a basic visual processing task. Our primary findings indicated that infected patients exhibit abnormal activation in the right DLPFC, right FEF, and the posterior cingulate cortices. For the right DLPFC and FEF regions, the amplitude of activation in HIV-infected patients was correlated with neuropsychological performance on a relatively sensitive test for HAND, thus connecting imaging and behavioral measurements of abnormal brain function due to HIV-infection. Lastly, the current study is not without limitations. All of our patients were undergoing combination antiretroviral therapy, and the effects of therapy on the MEG signal are unknown. In addition, some of our patients had a history of drug or alcohol use, which may have had a long-term impact on cortical physiology that is not directly related to HIV. We also studied only older adults with HIV, given their underrepresented status, and our findings cannot be generalized to younger patients who have been infected for a shorter period of time. Finally, our sample size was also rather small compared to previous fMRI studies, although it is comparable to many MEG studies in psychiatry and neurology. Future studies should use larger samples and evaluate the effects of antiretroviral treatment and infection duration.
